# Clustering of Gastrointestinal Microorganisms in Human Stool Samples from Ghana

**DOI:** 10.3390/pathogens13070583

**Published:** 2024-07-15

**Authors:** Joy Backhaus, Simone Kann, Andreas Hahn, Felix Weinreich, Martin Blohm, Konstantin Tanida, Torsten Feldt, Fred Stephen Sarfo, Veronica Di Cristanziano, Ulrike Loderstädt, Stephan Ehrhardt, Stefanie Schoppen, Harry Tagbor, Hagen Frickmann, Kirsten Alexandra Eberhardt

**Affiliations:** 1 Statistical Consulting, 97074 Wuerzburg, Germany; statistik.backhaus@googlemail.com; 2 Department of Microbiology and Hospital Hygiene, Bundeswehr Central Hospital Koblenz, 56070 Koblenz, Germany; simone_kann@hotmail.com; 3 Institute of Medical Microbiology, Immunology and Parasitology (IMMIP), University Hospital Bonn, 53127 Bonn, Germany; 4 Institute for Medical Microbiology, Virology and Hygiene, University Medicine Rostock, 18057 Rostock, Germany; andreas.hahn@uni-rostock.de; 5 German-Dutch Corps, 48143 Münster, Germany; felixweinreich@bundeswehr.org; 6 Laboratory Department, Bundeswehr Hospital Berlin, 10115 Berlin, Germany; martin2blohm@bundeswehr.org; 7 Institute for Microbiology, Virology and Hygiene, University Hospital Eppendorf, 20251 Hamburg, Germany; konstantin.tanida@gmail.com; 8 Department of Gastroenterology, Hepatology and Infectious Diseases, Medical Faculty and University Hospital Düsseldorf, Heinrich Heine University Düsseldorf, 40225 Düsseldorf, Germany; torsten.feldt@med.uni-duesseldorf.de; 9 Department of Medicine, Kwame Nkrumah University of Science and Technology, Kumasi 00233, Ghana; stephensarfo78@gmail.com; 10 Department of Medicine, Komfo Anokye Teaching Hospital, Kumasi 00233, Ghana; 11 Institute of Virology, Faculty of Medicine and University Hospital of Cologne, University of Cologne, 50935 Cologne, Germany; veronica.di-cristanziano@uk-koeln.de; 12 Institute for Infection Control and Infectious Diseases, University Medical Center Göttingen, 37075 Göttingen, Germany; ulrike.loderstaedt1@med.uni-goettingen.de; 13 Department of Epidemiology, Johns Hopkins Bloomberg School of Public Health, Baltimore, MA 21205, USA; sehrhar6@jhu.edu; 14 Department of Health and Social Science, Hochschule Fresenius, 20148 Hamburg, Germany; stefanie.schoppen@hs-fresenius.de; 15 School of Medicine, Department of Community Health, University of Health and Allied Sciences, Ho PMB 31, Ghana; htagbor@uhas.edu.gh; 16 Department of Microbiology and Hospital Hygiene, Bundeswehr Hospital Hamburg, 20359 Hamburg, Germany; 17 Department of Tropical Medicine, Bernhard Nocht Institute for Tropical Medicine & I. Department of Medicine, University Medical Center, 20359 Hamburg, Germany; 18 Division of Hygiene and Infectious Diseases, Institute of Hygiene and Environment, 20539 Hamburg, Germany

**Keywords:** cluster analysis, etiological relevance, gastrointestinal infections, high-prevalence setting, pathogen, commensal

## Abstract

The study was conducted to identify cluster patterns of enteric microorganisms with potential etiological relevance for infectious gastroenteritis in stool samples of individuals from Ghana, which is a known high-endemicity setting for infectious gastroenteritis. These patterns were compared to previous observations with specimens from Colombian indigenous people in order to assess potentially stable clustering for temporally and spatially distinct populations from high-endemicity regions. By doing so, the study aimed to identify stable clusters as markers of microbial interaction with potential importance for etiological relevance assignment in cases of multiple enteric pathogen detections. Stool samples from 1569 Ghanaian individuals (875 from HIV patients, 30 from HIV-negative control adult patients, and 644 from children < 2 years of age) were assessed for enteric microorganisms by applying real-time PCR. As a result, nucleic acids of bacterial microorganisms were most frequently detected, followed by protozoa, microsporidia, and helminths. Interestingly, the cluster assessment confirmed interaction patterns known from the previous analysis with Colombian indigenous people, demonstrating a high likelihood of *Blastocystis hominis* for clustering with other microorganisms and a prominent, potentially mediating role of *Dientamoeba fragilis* for microbial interactions within the clusters. In conclusion, the assessment confirmed conserved clustering of enteric microorganisms with potential etiological relevance for human infectious gastroenteritis over geographically distinct high-endemicity settings. Furthermore, the composition of abundant microorganisms is more important than regional factors for the determination of the interplay of enteric microorganisms in the human gut. Thereby, some microbial pathogens and commensals seem more susceptible to a changing microbial composition in the human gut than others.

## 1. Introduction

When gastrointestinal pathogens are detected in stool samples of patients with infectious gastroenteritis living in regions where such pathogens as well as associated infectious gastroenteritis are frequent, it can be difficult to say whether the detected pathogen is really the cause of the observed clinical symptoms. This is particularly true if more pathogens than just a single one are detected in the same stool sample. The problem that most enteric pathogens in regions with high prevalence for infectious gastroenteritis can both cause enteric disease or just persist as harmless colonizers is called “facultative pathogenicity” [1,2,3,4,5]. Previously, other researchers have tried to link the quantity of pathogens in stool samples with their likelihood of causing infectious gastroenteritis in such patients. In the case of diagnostic real-time PCR, so-called cycle threshold (Ct) values are an indirect option for target quantification because low Ct values indicate high quantities of the PCR target and vice versa. However, such attempts at linking pathogen quantity in stool samples with the likelihood of this pathogen causing clinical disease were only partly successful [2,3,6]. Consequently, generally accepted cut-offs for a Ct-value-based assignment of etiological relevance of enteric pathogens do not exist.

Obviously, more factors influence the likelihood of an association between clinically observed infectious gastroenteritis and the detection of an enteric pathogen in a human stool sample. One of these factors is “semi-immunity”, which means immunological adaptation of the human gut to rapid cycles of repeated pathogen exposure under poor hygiene conditions. This has been observed repeatedly in resource-limited tropical regions [7,8,9]. Although it is not yet completely understood how enteric semi-immunity works on a cellular or molecular level, some well-defined hypotheses regarding host–pathogen coexistence have been proposed, particularly for enteric helminth infections, as summarized elsewhere [10]. Furthermore, the semi-immunity concept is already in preventive medical use. Oral typhoid fever vaccination is the most commonly known example of induced short-term immunity on enteric mucous membranes [11].

Next to immunological adaptations, the enteric microbiome’s composition is believed to play a role in the degree of susceptibility towards the virulence of enteric pathogens. For both laboratory animals and human individuals, favorable microbiome compositions have been demonstrated to mitigate colonization resistance towards enteric pathogens, thus making gastroenteric infections less likely [12,13,14]. In addition, there is an increasing body of evidence suggesting likely interaction between enteric pathogens and commensals, which affects the clinical outcome [5,15].

In order to contribute to deciphering such microbial interactions in the gut of individuals from high-endemicity settings for infectious gastroenteritis, our study group recently conducted a cluster analysis assessing gastroenteric pathogens in stool samples of a Colombian indigenous population [16]. Within this population, a cluster consisting of *Blastocystis hominis*, *Campylobacter* spp., and *Giardia duodenalis* was shown to interact with *Dientamoeba fragilis* and *Ascaris lumbricoides* in a microbial-density-dependent way [16]. During the interpretation of this finding [16], however, it remained uncertain whether this observation just represented a regional peculiarity or a general pattern of microbial interaction in the human gut.

As such, it seemed promising to repeat the analysis with populations from other tropical high-endemicity settings for gastroenteric infections. Ghana, in the West African region, is an example of a country where enteric pathogens can be detected both in association with diarrheal disease and in asymptomatic individuals [4,17]. Especially high detection rates of gastroenteric pathogens can be expected in Ghanaian children [18,19,20], and in older studies, when diagnostic approaches with poor diagnostic accuracy, like Widal testing, were still in use [21], even underestimations of the true prevalence were likely. In such populations, co-occurrence of various microbial agents in stool samples as well as rapid pathogen acquisition cycles have been reported previously [22,23]. Furthermore, the common co-occurrence of genetic resistance determinants in Ghanaian stool samples [24,25] bears the risk of resistance transmission to bacterial enteric pathogens via mobile genetic elements. For *Campylobacter* spp. detections in Ghana, resistance was pronounced in cases of HIV-positive patients [26]. Enteric protozoan parasite infections of the gut have been reported to be particularly frequent in Ghanaian patients with diabetes [27]. Ghanaian farm environments provide reservoirs for several enteric pathogens like, e.g., salmonellae [28] and *Cryptosporidium* spp. [29]. Fecal contamination of regional environments is common [30], and food-borne or water-borne transmissions of infectious gastroenteritis are frequent events, particularly for poor Ghanaians [31,32,33]. Consequently, evidence of long-term efficiency of water filtration has recently been confirmed for Ghana [34], and, in line with the abovementioned information, colonized food vendors play a relevant role for such food-borne transmission events in Ghana [35].

Based on such previous experiences, Ghana was chosen as a suitable candidate region for cross-checking the experience from Colombia [16] in another tropical high-endemicity setting. To do so, cluster analyses were performed both with the set of microbial parameters chosen for the Colombian assessment [16] alone as well as in comparison with the Colombian results published elsewhere [16] and also with a broadened dataset available for the Ghanaian samples only. Furthermore, the dataset on Ghanaian individuals was sub-divided into subsets comprising children under 2 years of age and HIV-positive individuals. The rationale of these analytical steps is as follows. If microbial interactions are stable, comparable clustering should appear despite regionally different populations and despite interindividual differences, including factors like medical conditions and environmental factors. In summary, the study aimed at identifying stable clusters as markers of microbial interaction with potential importance for etiological relevance assignment in cases of multiple enteric pathogen detections, and the inclusion of different subpopulations, including children and HIV-positive individuals, was performed to further challenge the stability of potentially observed clustering.

## 2. Materials and Methods

### 2.1. Study Type

The study was conducted as a modelling approach using diagnostic real-time PCR data obtained from cross-sectional assessments of stool samples acquired from Ghanaian populations. It included a comparison of the Ghanaian results with historic data from a population of Colombian indigenous individuals [16].

### 2.2. Study Populations and Inclusion and Exclusion Criteria

The study population included a total of *n* = 1569 stool samples collected from Ghanaian individuals. The included subgroups comprised samples from *n* = 875 non-age-stratified Ghanaian HIV (human immunodeficiency virus) patients and *n* = 30 Ghanaian control individuals without known HIV infection [36] as well as from *n* = 664 Ghanaian children < 2 years of age.

The female:male ratio was 3:1. In the adult population (HIV-positive individuals and controls), the mean age (±standard deviation (SD)) was 39.6 (±9.9). Included Ghanaian HIV patients showed a median CD4+ T-cell count/μL (interquartile range (IRQ)) of 392.5 (189, 610) and a median CD4+/CD8+ T-cell ratio (IRQ) of 0.4 (0.2, 0.7). The median viral load in log10 copies/mL (IRQ) was 4.0 (1.6–5.2).

If sample material was insufficient for all real-time PCR assessments, this was not an exclusion criterion, and at least the available parameters were assessed. Samples showing inhibition of molecular diagnosis in the inhibition control PCR as detailed below were considered non-interpretable in cases lacking a positive PCR signal and positive in cases with an abundance of a PCR signal for a specific parameter. The following pathogens contained in the former study on Colombian indigenous individuals [16], from which data were used for comparison purposes, were not included in mathematical assessments: *Aeromonas* spp., *Trichuris trichiura*, and *Hymenolepis nana*. *Aeromonas* spp. was not tested with the Ghanaian samples, *Trichuris trichiura* was tested but it never occurred, and *Hymenolepis nana* was detected only once. To have comparable proportions, microorganisms had to appear with a prevalence of at least 1:100 (1%), constituting approximately a minimum of 6 in children and 10 in adults [16,37]. Applying this exclusion criterion, the following microorganisms were excluded from further analyses in the entire sample: *Hymenolepis nana*, *Necator americanus*, *Ascaris lumbricoides,* and *Taenia solium.* However, because *Taenia solium* was not differentiated from *Taenia saginata* in the Colombian assessment [16], detections of any of the two *Taenia* species were fused for the comparison of the Colombian dataset [16] with the Ghanaian PCR results.

### 2.3. Real-Time PCR Diagnostics

Stool samples were stored at −80 °C after sampling until nucleic acid extraction was conducted. Nucleic acids were extracted using the QIAamp stool DNA mini kit (Qiagen, Hilden, Germany). Real-time PCR was conducted by applying previously published protocols, as summarized in the following. Regarding the assessed bacterial microorganisms, the protocol by Wiemer et al. [38] was used for the detection of *Salmonella* spp. (*ttrC* sequence), *Shigella* spp./enteroinvasive *Escherichia coli* (EIEC, *ipaH* sequence), *Campylobacter jejuni* (*gyrA* sequence), and *Yersinia* spp. (*ail* sequence). The protocol by Hahn et al. [39] was used for enteropathogenic *Escherichia coli* (EPEC, EAF plasmid and *eae* sequences), enterotoxigenic *Escherichia coli* (ETEC, *eltB* and *estB* sequences), and enteroaggregative *Escherichia coli* (EAEC, *aatA* sequence). The protocol by Fenollar et al. [40] was used for *Tropheryma whipllei* (*Dig 15* sequence). For the diagnosis of EPEC and ETEC, a positive reaction with at least one of the target sequences was demanded to consider the sample as positive for the respective *Escherichia coli* pathovar. For protozoan parasites, the real-time PCR protocols by Verweij et al., Köller et al., ten Hove et al., and Stensvold et al. [41,42,43,44,45,46] were applied for the diagnosis of *Entamoeba histolytica* (SSU rRNA sequence), *Giardia duodenalis* (SSU rRNA sequence), *Cyclospora cayetanensis* (SSU rRNA sequence), *Cryptosporidium parvum* (138-bp fragment inside of the *C. parvum*-specific 452-bp fragment), *Cystoisospora belli* (ITS-2 sequence), *Dientamoeba fragilis* (5.8S rRNA sequence), and *Blastocystis hominis* (SSU rRNA sequence). For helminths, the real-time PCR protocols by Köller et al., Basuni et al., Praet et al., Obeng et al., and Kaisar et al. [42,47,48,49,50] were performed to diagnose *Ascaris lumbricoides* (ITS-1 sequence), *Ancylostoma* ssp. (ITS-2 sequence), *Necator americanus* (ITS-2 sequence), *Strongyloides stercoralis* (SSU rRNA sequence), *Taenis solium* (ITS-1 sequence), *Taenia saginata* (ITS-1 sequence), *Schistosoma* spp. (ITS-2 sequence), *Trichuris trichiura* (SSU rRNA sequence), *Enterobius vermuicularis* (ITS-1 sequence), and *Hymenolepis nana* (ITS-1 sequence). Finally, the real-time PCR protocol by Tanida et al. [51] was used for the diagnosis of microsporidia (SSU rRNA sequence of *Enterocytozoon bieneusi*, *Encephalcytozoon cuniculi*, *Encephalcytozoon hellem,* and *Encephalcytozoon intestinalis*). Sample inhibition was controlled using a real-time PCR targeting a sequence fragment of Phocid Herpes Virus (PhHV), as previously described by Niesters [52]. Therefore, from a total of 1569 assessed Ghanaian samples, 1496 (95.8%) did not show relevant sample inhibition in the PhHV-sequence-based inhibition control PCR. The applied oligonucleotides for the real-time PCR reactions are presented to interested readers in Appendix A Table A1. All assays were run on either RotoGene Q (Qiagen, Hilden, Germany) or MIC (Bio Molecular Systems, Upper Coomera, Australia) cyclers with plasmid-based positive controls and PCR-grade water-based negative controls in each run. Detection limits for the various assays ranged between 10^2^ and 10^4^ DNA copies per µL samples.

### 2.4. Statistical Assessment

Statistical analyses were carried out using the R 3.6.1 packages dplyr 2.3.0, fpc2.2-10, mclust 6.0.0, vegan2.6-4, dendextend 1.17.1, and ggplot2 3.4.2. Searching for clusters in the real-time PCR data was conducted using agglomerative hierarchical clustering with z-standardization [53]. Hierarchical clustering maximizes intra-class similarity and inter-class dissimilarity, which means pathogens within a cluster are algorithmically aligned to be similar and distinct from pathogens of other clusters. Cycle threshold (Ct) values of real-time PCR, which provide a semi-quantification approach, were clustered using the complete-linkage method to find an optimal solution in Euclidean space [54]. The Average Jaccard Index using 10.000 bootstrap resamples [55] was used to evaluate the stability of clusters, with values < 0.6 considered unstable, values ranging from 0.6 to 0.85 considered stable, and values greater 0.85 considered highly stabile [56]. The analysis included three major steps:

1. Native cluster analysis for all microorganisms eligible for the Ghanaian population.
2. Cluster analysis for microorganisms already included in the Colombian study [16] but with Ghanaian data to inspect interactions within a comparable composition of pathogens.
3. Direct comparison employing both the Ghanaian data and the original data from the Colombian study [16] using a tanglegram.

The tanglegram was detangled using the step2side algorithm, which facilitates visual comparison of two hierarchical dendrograms [57]. A cophenetic correlation matrix [58] was computed to compare statistical similarity between dendrograms [59]. Values close to zero were considered to represent no similarity, while values greater five were considered to represent moderate to high similarity between distance matrices.

### 2.5. Ethics

Ethical clearance for the assessments delivering the study data was obtained from the Committee on Human Research of the Kwame Nkrumah University of Science and Technology in Kumasi, Ghana, CHRPE/AP/12/11 and CHRPE/KNUST/KATH/01_06_08, and from the ethics committee of the Medical Council in Hamburg, Germany, under the reference numbers PV3771 and PV3020. The work was conducted in line with the Declaration of Helsinki and all of its amendments. Informed consent was provided by the study participants, or, in the case of minors, by their parents or next of kin.

## 3. Results

### 3.1. Summary of the Diagnostic Results

DNA of bacterial microorganisms was most frequently detected, followed by protozoa, microsporidia, and helminths. As shown in detail in Table 1, detections in declining order of frequency comprised enteropathogenic *Escherichia coli* (EPEC), enteroaggregative *E. coli* (EAEC), enterotoxigenic *E. coli* (ETEC), *Shigella* spp./enteroinvasive *E. coli* (EIEC), *Tropheryma whipplei*, *Salmonella* spp., *Campylobacter jejuni*, and *Yersinia* spp. among the assessed bacteria, *Blastocystis hominis*, *Giardia duodenalis*, *Cyclospora cayetanensis, Cryptosporidium parvum* (same frequency of the latter two microorganisms), *Entamoeba histolytica,* and *Cystoisospora belli* among the assessed protozoa, and, finally, *Schistosoma* spp., *Strongyloides stercoralis*, *Taenia saginata*, *Necator americanus, Taenia solium* (same frequency of the latter two microorganisms), *Ascaris lumbricoides,* and *Hymenolepis nana* among the assessed helminths.

Minor differences in the distribution of assessed microorganisms were seen over the different subpopulations of the study, as shown in Table 1; however, these differences mostly affected microorganisms generally detected in low numbers only. Of note, *Necator americanus* and *Taenia saginata* did not occur in the subpopulation of Ghanaian children. For the older subpopulations, *Hymenolepis nana* was not detected, and, in the children, only a single detection was recorded.

*Trichuris* spp. was included in PCR screening but excluded from the calculations described below due to lack of detection. Similarly, *Ancylostoma* spp. was not detected.

### 3.2. Cluster Calculations

Based on the predefined inclusion criteria (please also see paragraph 2.2 above for details), *n* = 19 microorganisms could be subjected to cluster analysis for the entire Ghanaian population, *n* = 17 for the Ghanaian HIV-positive subpopulation, and *n* = 14 for the Ghanaian children. Details are provided in Table 2.

**Figure 1:**
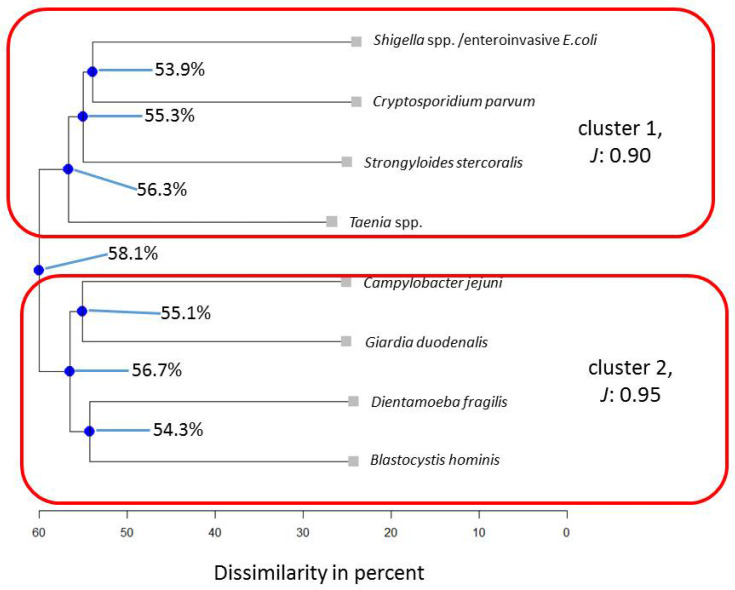
Results for microorganisms in the total Ghanaian population for the same parameters that had been assessed in indigenous Colombians [16]. Note: blue nodes represent connection points. *J* = Average Jaccard Index.

To potentially falsify the generalizability of cluster results found for a Colombian indigenous population in a previous assessment [16], in a first step, the following eight microorganisms were subjected to cluster analysis for the entire Ghanaian population: *Giardia duodenalis*, *Blastocystis hominis*, *Campylobacter jejuni*, *Dientamoeba fragilis*, *Strongyloides stercoralis*, *Cryptosporidium parvum*, *Shigella* spp./enteroinvasive *Escherichia coli*, and *Taenia* spp. (*Taenia saginata* and *Taenia solium*, compare with the methods section for details). Stratified for the subpopulation of Ghanaian HIV patients, seven microorganisms could be included, namely *Giardia duodenalis*, *Blastocystis hominis*, *Campylobacter jejuni*, *Strongyloides stercoralis*, *Cryptosporidium parvum*, *Shigella* spp./enteroinvasive *Escherichia coli,* and *Taenia* spp. For children, six microorganisms, i.e., *Giardia duodenalis*, *Blastocystis hominis*, *Campylobacter jejuni*, *Dientamoeba fragilis*, *Cryptosporidium parvum*, and *Shigella* spp./enteroinvasive *Escherichia coli,* could be included in this comparison. The Average Jaccard Index (*J*) ranged from 0.64 to 0.95, yielding stable to very stable results for the three sub-analyses. For the subpopulation of HIV patients, the Average Jaccard Index fell below the cut-off value of 0.60 (*J* = 0.54) for one cluster. Most stable results were determined for the Ghanaian cluster solution based on the composition found in indigenous Colombian individuals (*J* = 0.95, Figure 1).

Within this assessment, the top node merges at 58.1, indicating that in 41.9% of cases, cluster 1 and cluster 2 interact in a similar pattern. *Shigella* spp./enteroinvasive *Escherichia coli* and *Cryptosporidium parvum*, *Campylobacter jejuni,* and *Giardia duodenalis* as well as *Dientamoeba fragilis* and *Blastocystis hominis* show similar behavior in approximatively 45% of cases within their cluster in the abundance of co-modulating others.

**Figure 2:**
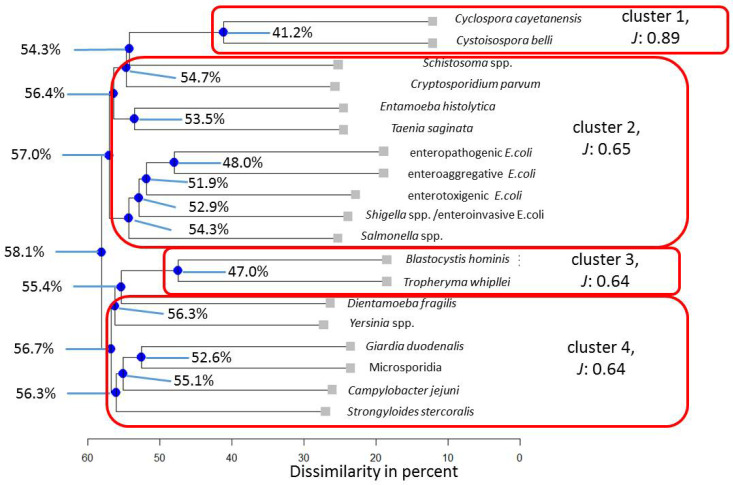
Cluster analytical results for the entire Ghanaian population. Note: blue nodes represent connection points. *J* = Average Jaccard Index.

For the Ghanaian assessment comprising 19 microorganisms for the total population, a four-cluster solution described the observed data best (Figure 2). As indicated by the Average Jaccard Index, *Cyclospora cayetanensis* and *Cystoisospora belli* show similar interaction, accounting for a predictable pattern in 58.8% of cases.

**Figure 3:**
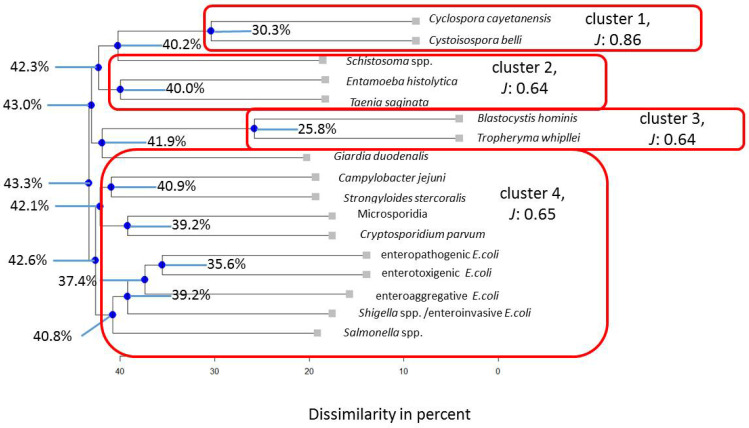
Results for the HIV-positive Ghanaian subpopulation. Note: blue nodes represent connection points. *J* = Average Jaccard Index.

**Figure 4:**
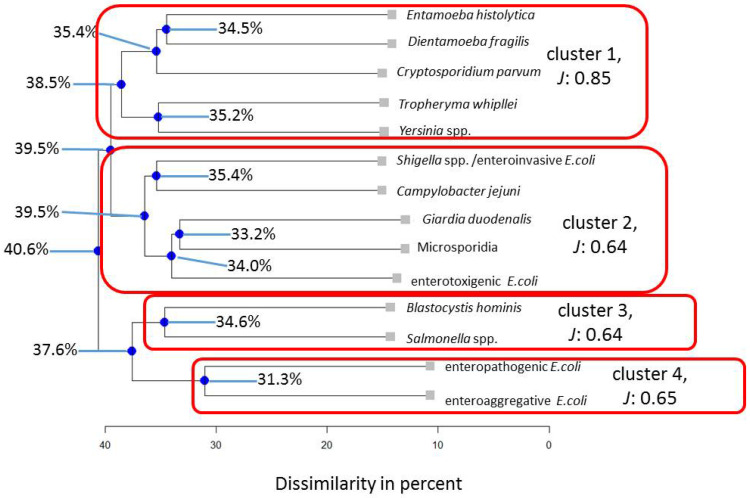
Results for the Ghanaian children subpopulation. Note: blue nodes represent connection points. *J* = Average Jaccard Index.

Similarly, four clusters described the subpopulations stratified by HIV positivity (Figure 3) and being children < 2 years of age best (Figure 4). In the HIV-positive subpopulation, the interaction pattern between *Blastocystis hominis* and *Tropheryma whipplei* is similar in 74.2% of cases, while this value is 53% for the entire Ghanian population. Therefore, the low values for the nodes of *Cystoisospora belli* and *Cyclospora cayetanensis* as well as *Tropheryma whipllei* and *Blastocystis hominis* indicate that within this clustering, both tend to preferentially occur in these dual associations. In children, *Giardia duodenalis* aligns close to micosporidia (66.8%), which is similar in the adult sample but not as pronounced (47.4%). In the HIV-positive subpopulation, this association disappears.

Focusing on matching or mismatching results compared to the previous Colombian analysis [16], computing the cluster analysis for microbial parameters also assessed in the sample of indigenous Colombians [16] for the here-presented Ghanaian population indicated that a two-cluster solution describes the observed data best (Figure 1).

**Figure 5:**
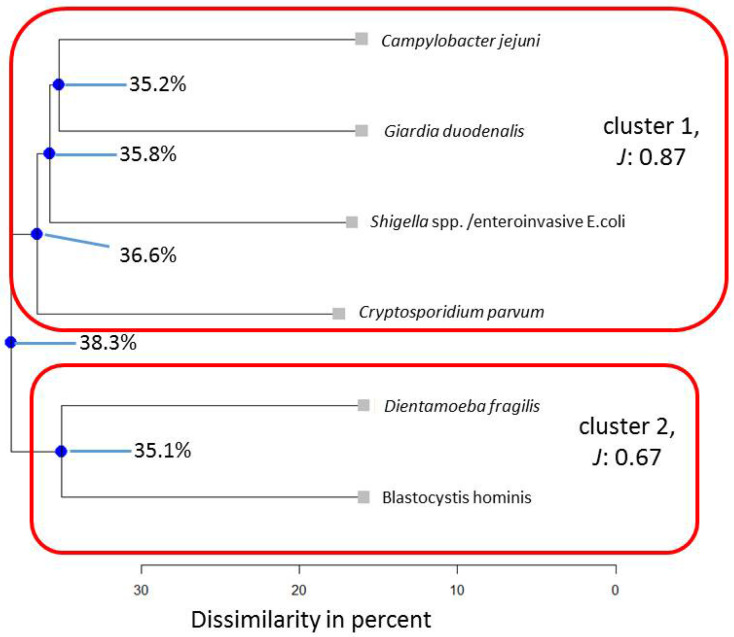
Results for the Ghanaian children subpopulation for the same parameters that had been assessed in indigenous Colombians [16]. Note: blue nodes represent connection points. *J* = Average Jaccard Index.

Therefore, *Blastocystis hominis* tends to form close binary associations, and this tendency stays stable even when the composition of microorganism varies. In particular, *Blastocystis hominis* and *Dientamoeba fragilis* align together when assessing all adults (45.7%) as well as all children from the Ghanaian population (64.9%, cf. Figure 5).

**Figure 6:**
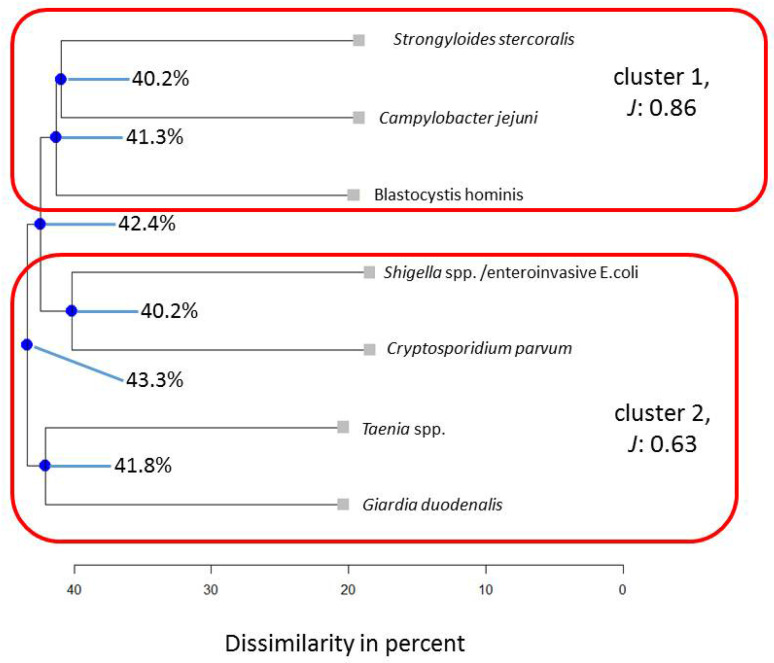
Results for the Ghanaian HIV-positive subpopulation for the same parameters that had been assessed in indigenous Colombians [16]. Note: blue nodes represent connection points. *J* = Average Jaccard Index.

In HIV patients, however, the latter observation could not be made, because *Dientamoeba fragilis* was recorded only twice in this subpopulation and, consequently, did not meet the inclusion criteria for the cluster analysis (Figure 6).

When occurring together in the stool samples, *Blastocystis hominis* and *Dientamoeba fragilis* correlated as highly positive (*r* = 0.78, *p* < 0.05). In contrast, when *Dientamoeba fragilis* was absent, real-time PCR cycle threshold (Ct) values for *Blastocystis hominis* were significantly lower (18.6 (±18.1), indicating higher microbial loads) compared to the co-abundance of *Dientamoeba fragilis* (32.8 (±6.2)), *p* < 0.05), a pattern already seen for indigenous Colombians [16]. As indicated in Figure 6, *Cryptosporidium parvum* and *Shigella* spp./enteroinvasive *Escherichia coli* form a stable association across stratifications for the microbial composition, as previously investigated in indigenous Colombians [16], while the configuration changes slightly in Ghanaian children (Figure 5).

**Figure 7:**
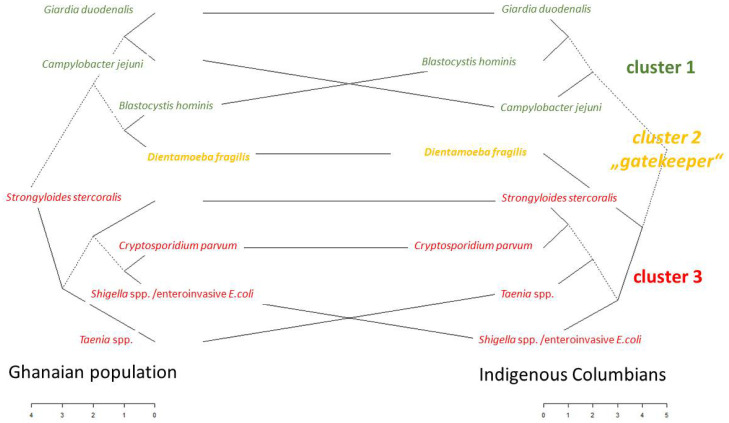
Tanglegram for the cluster solution based on data from the Ghanaian population presented here and using data from indigenous Colombians (published as [16]). Dotted lines represent paths based on statistical interaction of multiple microorganisms.

Considering these descriptive similarities for the now-assessed Ghanaian and the previously assessed Colombian population [16], a tanglegram for direct inferential comparison was computed (Figure 7). Therefore, data from the former study on indigenous Colombians [16] were directly compared to the entire Ghanaian population. A cophenetic correlation of 0.52 demonstrated moderate to high inter-cluster stability, thus indicating similarity between both populations.

In this direct comparison of the geographically distinct populations, there are nevertheless a few peculiarities. In particular, and as visualized in Figure 7, *Dientamoeba fragilis* switches from a direct association with cluster 1 of the Ghanaian population in the direction of cluster 3 of the previously assessed indigenous Colombian population [16]. Of note, there are some branches in the tanglegram deserving particular notice. *Dientamoeba fragilis* appears in both populations at a prominent position prior to contact of cluster 1 microorganisms with cluster 3 microorganisms in the tanglegram in Figure 7, a mechanism that had already been described as a “gatekeeper” function for the Colombian population [16]. Furthermore, in both populations, *Taenia* spp. has contact with *Dientamoeba fragilis* before *Strongyloides stercoralis* and *Cryptosporidium parvum* fuse in the tanglegram. The proximity of *Shigella* spp./enteroinvasive *Escherichia coli* and *Cryptosporidium parvum*, in contrast, is different upon comparing the Ghanaian and the Colombian populations.

## 4. Discussion

This study was conducted to assess enteric microbial clustering in Ghanaian individuals and to compare these findings to previously published results from a geographically distinct population of Colombian indigenous individuals [16] and thus from another high-endemicity setting for gastrointestinal pathogens. The study led to a number of findings.

Focusing on previous experience with the molecular assessment of stool samples from Ghanaian patients with and without infectious gastroenteritis [4], the quantitative dominance of bacteria followed by protozoa as observed in the present investigation is not surprising. Also, the recorded low prevalence of enteric helminths in the Ghanaian stool samples compared to the previously assessed population of Colombian indigenous individuals [16] is well in line with another comparable Ghanaian publication [60].

Remarkable matching was observed regarding the cluster compositions of enteric microorganisms, as calculated for the presently described Ghanaian and the previously assessed Colombian populations [16], in spite of temporal and spatial distinctions. This particularly applies to the *Blastocystis–Campylobacter–Giardia* cluster and the prominent role of *Dientamoeba fragilis* in the cluster composition and likely also to the cluster interaction, as observed for the Colombian indigenous people before [16]. This stability is more interesting, considering the surprisingly low prevalence of *Dientamoeba fragilis* in the Ghanaian HIV patients, although HIV infections are generally considered to facilitate enteric colonization with *Dientamoeba fragilis* [61]. It may be speculated that commonly applied anti-helminthic treatment with benzimidazoles [60], which most likely also accounts for the low prevalence of helminths in the Ghanaian population, might have led to low *Dientamoeba fragilis* prevalence as well. Furthermore, it is interesting that the clustering remains stable considering the low prevalence of *Campylobacter jejuni* in the Ghanaian stool samples, both compared to the situation in Colombia [16] and previous Ghanaian investigations [4,26,62]. In contrast, minor differences between the Ghanaian and the Colombian populations, like, e.g., those observed for *Shigella* spp./enteroinvasive *Escherichia coli* and *Cryptosporidium parvum*, might be well-explained by the differential effects of varying prevalence and varying microbial compositions. Recently, of note, associations of the composition of the enteric microbiome both with the persistence of hookworms in spite of albendazole treatment [63] and with varying virulence of enteroaggregative *Escherichia coli* [64] have been proposed by Ghanaian researchers. In any case, it is interesting that the co-phrenic correlation coefficient is just close to 0.5 in spite of the pronounced similarity of the Ghanaian and the Colombian matrices in the tanglegram. This indicates that factors not assessed in the here-presented holistic approach are likely to be of relevance. This is particularly interesting considering the mentioned minor differences in the stratified cluster analyses with the Ghanaian subpopulations.

The methodical issues for the present analysis deserve critical consideration as well. The present approach utilized hierarchical clustering in order to verify or falsify findings of the prior cluster analysis with the Colombian specimens [16]. Considering the current opinion on statistical findings related to cluster analysis, fuzzy clustering might be an alternative appropriate approach. This is particularly the case because of the naturally varying subject-to-variables ratio in in the current study [37] and may be considered as a potential limitation of the assessment.

Furthermore, based on the knowledge gained from the previous cluster analysis of indigenous Colombians [16], the interaction between *Blastocystis hominis* and *Dientamoeba fragilis* was put into focus in the present assessment of reproducibility. This approach was justified by the most likely reasonable attempt of beginning to focus on the analysis of those microorganisms that align in face of different co-occurring microorganisms and subpopulations. However, thorough (non-)linear analysis of interactions indicated by our results between these two and all other possible combinations is required to achieve a more comprehensive pattern analysis in future study approaches. For example, the analysis has shown that *Cystoisospora belli* and *Cyclospora cayetanensis* as well as *Tropheryma whipplei* and *Blastocystis hominis* align together. Unfortunately, it was beyond the scope of this assessment to scrutinize their interplay more closely. Another potentially interesting association arising from close inspection of dendrograms is the close alignment of microsporidia and *Giardia duodenalis* in Ghanaian children, while this association at least partially loosens in HIV-positive adult individuals. In the latter subpopulation, microsporidia detections are more likely to be of etiological relevance [51], a factor potentially negatively interfering with otherwise facilitating effects of microsporidia on the abundance of *Giardia duodenalis* and vice versa.

When critically reflecting on the chosen methodology, it also needs to be addressed that the comparison using a tanglegram bears optimization problems that may be caused by the step2 algorithm used [65]. Exploratory variation of algorithms implemented in the R-package dendextend, however, verified the solution proposed to a great extent. As such, we feel justified to assume the validity of the approach. Another undeniable limitation of the assessment comprises the limited sample count considering the complexity of the assessed interactions and the rarity of some of the measured parameters. Due to logistic reasons and funding restraints, however, a broadening of the assessment was unfeasible. Broader assessments, however, need to be considered if underlying patterns of likely symbiosis between pathogens in the presence or absence of others shall be addressed in more detail in future confirmatory studies.

## 5. Conclusions

Despite the abovementioned limitations, the study indicates conserved clustering of enteric microorganisms with potential etiological relevance for human infectious gastroenteritis in high-endemicity settings. Furthermore, the analysis suggests that it is more the composition of abundant microorganisms rather than other regional factors that determines the interplay of enteric microorganisms in the assessed individuals’ gut. Therefore, some microbial pathogens and commensals seem more susceptible to a changing microbial composition in the human gut than others. Future assessment should aim at further addressing stable components within this complex interplay in order to better understand geographically varying susceptibility towards infectious gastroenteritis.

## Figures and Tables

**Table 1:** Diagnostic results obtained for the Ghana study population and its different subpopulations.

Parameter	Total Ghanaian Population (1569 Individuals), *n* (%), Mean Ct Value (±SD)	HIV-Positive Subpopulation (875 Individuals), *n* (%), Mean Ct Value (±SD)	Control Individuals for the HIV-Positive Subpopulation (30 Individuals), *n* (%), Mean Ct Value (±SD)	Subpopulation of Ghanaian Children < 2 Years of Age (664 Individuals), *n* (%), Mean Ct Value (±SD)
*Salmonella* spp. (*ttrC* sequence)	119 (7.6%), 29.8 (±3.0)	96 (10.7%), 29.6 (±3.1)	3 (10%), 28.0 (±2.0)	23 (3.5%), 31.0 (±2.1)
*Shigella* spp./enteroinvasive *Escherichia coli* (EIEC, *ipaH* sequence)	261 (16.7%), 27.6 (±5.8),	196 (21.8%), 26.7 (±6.0)	2 (6.7%), 31.5 (±0.7)	65 (9.8%), 30.3 (±4.2)
*Campylobacter jejuni* (*gyrA* sequence)	49 (3.1%), 29.6 (±6.2)	19 (2.1%), 28.1 (±6.4)	2 (6.7%), 32.5 (±6.4)	30 (4.5%), 30.6 (±5.9)
*Yersinia* spp. (*ail* sequence)	20 (1.3%), 34.5 (±2.7)	1 (0.1%) 36.0	0 (0%)	19 (2.9%) 34.4 (±2.7)
*Escherichia coli* (EPEC, based on EAF plasmid and/or *eae* sequence)	1012 (64.5%), 28.1 (±4.7)	605 (67.1%), 27.8 (±4.83)	28 (93.3%), 28.8 (±4.2)	407 (61.6%), 28.8 (±4.4)
enterotoxigenic *Escherichia coli* (ETEC, based on ST and/or LT sequences)	578 (37.9%), 30.4 (±4.7)	327 (36.3%), 30.4 (±4.2)	10 (33.3%), 29.8 (±5.9)	251 (38.0%), 30.3 (±5.2)
enteroaggregative *Escherichia coli* (EAEC, *aatA* sequence)	1009 (64.6%), 28.9 (±5.3)	636 (70.6%), 28.2 (±5.2)	17 (56.7%), 29.8 (±2.5)	373 (56.4%), 29.9 (±5.5)
*Tropheryma whipplei* (*Dig 15* sequence)	140 (9%), 34.9 (±2.1)	83 (9.2%), 35.3 (±2.2)	3 (10%), 37.7 (±0.6)	57 (8.6%), 34.3 (±1.8)
*Entamoeba histolytica* (SSU rRNA sequence)	66 (4.2%), 36.8 (±5.6)	54 (6.0%), 37.1 (±6.3)	1 (3.3%), 38.0, (-)	12 (1.8%), 35.5 (±7.2)
*Giardia duodenalis* (SSU rRNA sequence)	190 (12.2%), 30.8 (±4.5)	95 (10.5%), 30.4 (±4.2)	4 (13.3%), 29.5 (±5.7)	95 (14.4%), 32.4 (±3.5)
*Cyclospora cayetanensis* (SSU rRNA sequence)	71 (4.5%), 34.7 (±4.3)	63 (7.0%), 34.4 (±4.4)	1 (3.3%), 39.0, (-)	8 (1.2%), 36.9 (±2.0)
*Cryptosporidium parvum* (138-bp fragment inside of the *C. parvum*-specific 452-bp fragment)	71 (4.5%), 31.6 (±4.2)	56 (6.2%), 31.0 (±4.3)	0 (0%), n.a.	15 (2.3%), 33.8 (±2.5)
*Cystoisospora belli* (ITS-2 sequence)	34 (2.2%), 30.9 (±4.3)	33 (3.8%), 30.8 (±4.3)	0 (0%), n.a.	1 (0.2%), 35.0 (-)
*Dientamoeba fragilis* (5.8S rRNA sequence)	13 (0.8%), 32.7 (±5.7)	2 (0.2%), 39.0 (±4.2)	1 (3.3%), 36.0 (-)	11 (1.7%), 31.5 (±5.3)
*Blastocystis hominis* (SSU rRNA sequence)	356 (22.7%), 32.8 (±6.2)	106 (11.8%), 36.1 (±2.6)	3 (10%), 37 (±2.0)	250 (37.7%), 31.4 (±6.7)
*Ascaris lumbricoides* (ITS-1 sequence)	3 (0.2%), 25.7 (±3.5)	1 (0.1%), 22.0 (-)	0 (0%), n.a.	2 (0.3%), 27.5 (±2.1)
*Necator americanus* (ITS-2 sequence)	6 (0.4%), 36.2 (±2.9)	2 (0.7%), 36.2 (±2.9)	3 (10.0%), 37.3 (±2.1)	0 (0%), n.a.
*Strongyloides stercoralis* (SSU rRNA sequence)	18 (1.2%), 28.8 (±3.7)	17 (1.9%), 28.7 (±3.8)	0 (0%), n.a.	1 (0.2%), 31.0 (-)
*Taenis solium* (ITS-1 sequence)	6 (0.4%), 36.0 (±5.3)	5 (0.6%), 38.0 (±2.3)	0 (0%), n.a.	1 (0.2%), 26.0 (-)
*Taenia saginata* (ITS-1 sequence)	14 (0.9%), 36.5 (±3.0)	14 (1.6%), 36.5 (±3.0)	0 (0%), n.a.	0 (%), n.a.
*Schistosoma* spp. (ITS-2 sequence)	33 (2.1%), 28.3 (±6.2)	29 (2.8%), 25.0 (±5.6)	0 (0%), n.a.	8 (1.2%), 30.1 (±6.8)
*Hymenolepis nana* (ITS-1 sequence)	1 (0.1%), 35.0 (-)	0 (0%), n.a.	0 (0%), n.a.	1 (0.2%), 35.0 (-)
microsporidia (SSU rRNA sequence of *Enterocytozoon bieneusi*, *Encephalcytozoon cuniculi*, *Encephalcytozoon hellem,* and *Encephalcytozoon intestinalis*)	128 (8.2%), 28.2 (±5.9)	67 (7.4%), 25.5 (±5.4)	0 (0%), n.a.	61 (9.2%), 31.1 (±5.1)

ITS = internal transcribed spacer, rRNA = ribosomal ribonucleic acid, SSU = small subunit, Ct = cycle threshold, *n* = number, SD = standard deviation. n.a. = not applicable. (-) = lacking value.

**Table 2:** Microorganisms subjected to cluster analysis for the entire Ghanian population and the assessed subpopulations of HIV-positive individuals and children < 2 years of age based on the definitions from the methods section.

	Total Ghanaian Population	Subpopulation of HIV-Positive Individuals	Subpopulation of Children < 2 Years of Age
Number of included microbial parameters	*n* = 19	*n* = 17	*n* = 14
Details regarding included microbial parameters	*Dientamoeba fragilis*, *Yersinia* spp., *Strongyloides stercoralis*, *Campylobacter jejuni*, *Schistosoma* spp., *Cystoisospora belli*, *Entamoeba histolytica*, *Cryptosporidium parvum*, *Cyclospora cayetanensis*, microsporidia, *Tropheryma whipplei*, *Giardia duodenalis*, *Salmonella* spp., *Blastocystis hominis*, *Shigella* spp./enteroinvasive *Escherichia coli*, enterotoxigenic *Escherichia coli*, enteropathogenic *Escherichia coli,* and enteroaggregative *Escherichia coli*	*Taenia saginata*, *Strongyloides stercoralis*, *Campylobacter jejuni*, *Schistosoma* spp., *Cystoisospora belli*, *Entamoeba histolytica*, *Cyclospora cayetanensis*, *Cryptosporidium parvum*, microsporidia, *Tropheryma whipplei*, *Salmonella* spp., *Giardia duodenalis*, *Blastocystis hominis*, *Shigella* spp./enteroinvasive *Escherichia coli*, enterotoxigenic *Escherichia coli*, enteropathogenic *Escherichia coli*, enteroaggregative *Escherichia coli*	*Dientamoeba fragilis*, *Entamoeba histolytica*, *Cryptosporidium parvum*, *Yersinia* spp., *Salmonella* spp., *Campylobacter jejuni*, *Tropheryma whipplei*, microsporidia, *Shigella* spp./enteroinvasive *Escherichia coli*, *Giardia duodenalis*, *Blastocystis hominis*, enterotoxigenic *Escherichia coli*, enteroaggregative *Escherichia coli*, enteropathogenic *Escherichia coli*

*n* = numbers.

## Data Availability

All relevant data are provided in the manuscript, Appendix A Table A1, and its supporting information. Raw data can be made available upon reasonable request.

## Appendix A

**Table A1:** List of oligonucleotide sequences used for the real-time PCR assessments and their sources. Triplet structure of bases was just included to increase readability but does not indicate specific codons.

PCR Targets	Forward Primer Sequences	Reverse Primer Sequences	Probe Sequences	Sources
*Salmonella* spp. (*ttrC*)	ATT-GTT-GAT-TCA-GGT-ACA-AAC	AAT-TAG-CCA-TGT-TGT-AAT-CTC	CAA-GTT-CAA-CGC-GCA-ATT-TA	Wiemer et al., 2011 [38]
*Shigella* spp./enteroinvasive *Escherichia coli* (*ipaH*)	CAG-AAG-AGC-AGA-AGT-ATG-AG	CAG-TAC-CTC-GTC-AGT-CAG	ACA-GGT-GAT-GCG-TGA-GAC-TG	Wiemer et al., 2011 [38]
*Campylobacter jejuni* (*gyrA*)	CTA-TAA-CAA-CTG-CAC-CTA-CTA-AT	AAG-TGT-AAG-CAC-ACA-AGG-TA	CTT-AAT-AGC-CGT-CAC-CCC-AC	Wiemer et al., 2011 [38]
*Yersinia* spp. (*ail*)	GCA-TTA-ACG-AAT-ATG-TTA-GC	ATC-GAG-TTT-GGA-GTA-TTC-AT	CCG-CTT-CCA-AAT-TTT-GTC-AT	Wiemer et al., 2011 [38]
enteropathogenic *Escherichia coli* (*eae*; EAF plasmid sequence)	CAT-TGA-TCA-GGA-TTT-TTC-TGG-TGA-TA; CAG-GGT-AAA-AGA-AAG-ATG-ATA-A	CTC-ATG-CGG-AAA-TAG-CCG-TTA; GCA-TGG-AAC-ATC-GAT-CAG-TGA	ATA-GTC-TCG-CCA-GTA-TTC-GCC-ACC-AAT-ACC; TGG-AGT-GAT-CGA-ACG-GGA-TCC-A	Hahn et al., 2017 [39]
enterotoxigenic *Escherichia coli* (*eltB*, *estB*)	GCG-TTA-CTA-TCC-TCT-CTA-TG; TCC-CTC-AGG-ATG-CTA-AAC	TGA-TAT-TCC-GAA-CAT-AGT-TCT-GTA; CAA-CAA-AGC-AAC-AGG-TAC-ATA-CGT	TAG-ACT-GGG-GAG-CTC-CGT-GTG-C; ATA-GCA-CCC-GGT-ACA-AGC-AGG	Hahn et al., 2017 [39]
enteroaggregative *Escherichia coli* (*aatA*)	CAA-TGT-ATA-GAA-ATC-CGC-TGT-T	CTG-TCA-GAT-AAA-ATC-TCG-AGA-GAA	CAT-GTT-CCT-GAG-AGT-GCA-ATC-CCA-G	Hahn et al., 2017 [39]
*Tropheryma whipplei* (*Dig 15*)	TGT-TTT-GTA-CTG-CTT-GTA-ACA-GGA-TCT	TCC-TGC-TCT-ATC-CCT-CCT-ATC-ATC	AGA-GAT-ACA-TTT-GTG-TTA-GTT-GTT-ACA	Fenollar et al., 2008 [40]
*Entamoeba histolytica* (SSU rRNA)	ATT-GTC-GTG-GCA-TCC-TAA-CTC-A	GCG-GAC-GGC-TCA-TTA-TAA-CA	TCA-TTG-AAT-GAA-TTG-GCC-ATT-T	Verweij et al., 2004 [41]
*Giardia duodenalis* (SSU rRNA)	GAC-GGC-TCA-GGA-CAA-CGG-TT	TTG-CCA-GCG-GTG-TCC-G	CCC-GCG-GCG-GTC-CCT-GCT-AG	Verweij et al., 2004 [41]
*Cyclospora cayetanensis* (SSU rRNA)	TAG-TAA-CCG-AAC-GGA-TCG-CAT-T	AAT-GCC-ACG-GTA-GGC-CAA-TA	CCG-GCG-ATA-GAT-CAT-TCA-AGT-TTC-TGA-CC	Köller et al., 2020 [42], modified from Verweij et al., 2003 [43]
*Cryptosporidium parvum* (138-bp fragment inside of the *C. parvum*-specific 452-bp fragment)	CGC-TTC-TCT-AGC-CTT-TCA-TGA	CTT-CAC-GTG-TGT-TTG-CCA-AT	CCA-ATC-ACA-GAA-TCA-TCA-GAA-TCG-ACT-GGT-ATC	Verweij et al., 2004 [41]
*Cystoisospora belli* (ITS-2)	ATA-TTC-CCT-GCA-GCA-TGT-CTG-TTT	CCA-CAC-GCG-TAT-TCC-AGA-GA	CAA-GTT-CTG-CTC-ACG-CGC-TTC-TGG	ten Hove et al., 2008 [44]
*Dientamoeba fragilis* (5.8S rRNA)	CAA-CGG-ATG-TCT-TGG-CTC-TTT-A	TGC-ATT-CAA-AGA-TCG-AAC-TTA-TCA-C	CAA-TTC-TAG-CCG-CTT-AT	Verweij et al., 2007 [45]
*Blastocystis hominis* (SSU rRNA)	GGT-CCG-GTG-AAC-ACT-TTG-GAT-TT	CCT-ACG-GAA-ACC-TTG-TTA-CGA-CTT-CA	TCG-TGT-AAA-TCT-TAC-CAT-TTA-GAG-GA	Stensvold et al., 2012 [46]
*Ascaris lumbricoides* (ITS-1)	GTA-ATA-GCA-GTC-GGC-GGT-TTC-TT	GCC-CAA-CAT-GCC-ACC-TAT-TC	TTG-GCG-GAC-AAT-TGC-ATG-CGA-T	Basuni et al., 2011 [47]
*Ancylostoma* spp. (ITS-2)	GAA-TGA-CAG-CAA-ACT-CGT-TGT-TG	ATA-CTA-GCC-ACT-GCC-GAA-ACG-T	ATC-GTT-TAC-CGA-CTT-TAG	Basuni et al., 2011 [47]
*Necator americanus* (ITS-2)	CTG-TTT-GTC-GAA-CGG-TAC-TTG-C	ATA-ACA-GCG-TGC-ACA-TGT-TGC	CTG-TAC-TAC-GCA-TTG-TAT-AC	Basuni et al., 2011 [47]
*Strongyloides stercoralis* (SSU rRNA)	GAA-TTC-CAA-GTA-AAC-GTA-AGT-CAT-TAG-C	TGC-CTC-TGG-ATA-TTG-CTC-AGT-TC	ACA-CAC-CGG-CCG-TCG-CTG-C	Basuni et al., 2011 [47]
*Taenia solium* (ITS-1)	ATG-GAT-CAA-TCT-GGG-TGG-AGT-T	ATC-GCA-GGG-TAA-GAA-AAG-AAG-GT	TGG-TAC-TGC-TGT-GGC-GGC-GG	Praet et al., 2013 [48]
*Taenia saginata* (ITS-1)	GCG-TCG-TCT-TTG-CGT-TAC-AC	TGA-CAC-AAC-CGC-GCT-CTG	CCA-CAG-CAC-CAG-CGA-CAG-CAG-CAA	Praet et al., 2013 [48]
*Schistosoma* spp. (ITS-2)	GGT-CTA-GAT-GAC-TTG-ATY-GAG-ATG-CT	TCC-CGA-GCG-YGT-ATA-ATG-TCA-TTA	TGG-GTT-GTG-CTC-GAG-TCG-TGG-C	Obeng et al., 2008 [49]
*Trichuris trichiura* (SSU rRNA)	TTG-AAA-CGA-CTT-GCT-CAT-CAA-CTT	CTG-ATT-CTC-CGT-TAA-CCG-TTG-TC	CGA-TGG-TAC-GCT-ACG-TGC-TTA-CCA-TGG	Kaisar et al., 2017 [50]
*Enterobius vermicularis* (ITS-1)	CGG-TGT-AAT-TTT-GTT-GGT-GTC-TAT-G	TGG-CAG-CAT-TGC-AAA-CTA-ATG	TGT-GCC-AGT-CAA-CGC-CTA-AAC-CGT-C	Köller et al., 2000 [42]
*Hymenolepis nana* (ITS-1)	CAT-TGT-GTA-CCA-AAT-TGA-TGA-TGA-GTA	CAA-CTG-ACA-GCA-TGT-TTC-GAT-ATG	CGT-GTG-CGC-CTC-TGG-CTT-ACC-G	Köller et al., 2000 [42]
Microsporidia (SSU rRNA of *Enterocytozoon bieneusi*, *Encephalcytozoon cuniculi*, *Encephalcytozoon hellem* and *Encephalcytozoon intestinalis*)	CAC-CAG-GTT-GAT-TCT-GCC-TGA; TCC-GGA-GAG-GGA-GCC-TGA-G	GCT-TGC-CCT-CCA-ATT-GCT-TC; GAC-TTG-CCC-TCC-AAT-CAC-ATG; CCG-ACT-TGC-CCT-CCA-GTA-AA; CTT-GGC-CTC-CAA-TCA-ATC-TCG	TGG-CAG-CAG-GCG-CGA-AAC-TTG-T	Tanida et al., 2022 [51]
Internal control assay parameter Phocid Herpes Virus (PhHV) sequence	GGG-CGA-ATC-ACA-GAT-TGA-ATC	GCG-GTT-CCA-AAC-GTA-CCA-A	TTT-TTA-TGT-GTC-CGC-CAC-CAT-CTG-GAT-C	Niesters, 2001 [52]

ITS = internal transcribed spacer, rRNA = ribosomal ribonucleic acid, SSU = small subunit.
